# Implementing resilience-based interventions for healthcare employee well-being: evidence from the pandemic crisis

**DOI:** 10.3389/fpubh.2025.1606595

**Published:** 2025-08-25

**Authors:** Ewa Agnieszka Kosycarz, Dorota Juszczak, Monika Raulinajtys-Grzybek, Anna Krejner-Nowecka

**Affiliations:** ^1^Department of Economic Theory, Collegium of Socio-Economics, SGH Warsaw School of Economics, Warsaw, Poland; ^2^Department of Mathematical Statistics, Institute of Econometrics, Collegium of Economic Analysis, SGH Warsaw School of Economics, Warsaw, Poland; ^3^Department of Management Accounting, Collegium of Business Administration, SGH Warsaw School of Economics, Warsaw, Poland; ^4^Department of Personnel Strategies, Institute of Management, Collegium of Management and Finance, SGH Warsaw School of Economics, Warsaw, Poland

**Keywords:** organizational resilience, sense of security, stress caused by working conditions, well-being of healthcare workers, rewards and recognition

## Abstract

**Introduction:**

Organizational resilience is of paramount importance for coping with adversity, particularly in the healthcare sector during crises. The objective of the present study was to evaluate the impact of resilience-based interventions on the well-being of healthcare employees during the pandemic. In this study, resilience-based interventions are defined as organizational actions that strengthen a healthcare institution’s capacity to cope with crises—such as ensuring adequate personal protective equipment and staff testing, clear risk-communication, alternative care pathways (e.g., telemedicine) and psychosocial support—each mapping onto the recognized resilience capabilities of material resources, information management, collateral pathways and human-capital management The research question focused on two key aspects: first, whether Polish healthcare institutions effectively implemented these interventions, and second, how these interventions were perceived by their employees. The hypothesis tested was that resilience-based interventions positively influence employee well-being.

**Methods:**

The study was conducted between August 21, 2020, and October 6, 2020, in Poland (across all regions). It utilized a cross-sectional, online survey-based approach, targeting healthcare professionals. A 39-item questionnaire was developed and distributed via Microsoft Forms, with participants recruited through websites and newsletters from doctors, nurses, and midwives’ associations. A variety of statistical methods were used to analyze the obtained data, i.e., logistic regression, proportional ordinal logistic regression, multiple marginal independence test, simultaneous pairwise marginal independence test, Cochran Q test, random forest-based imputation of missing data.

**Results:**

The study found that resilience-based interventions, such as access to personal protective equipment and virus-detection testing, significantly reduced anxiety among healthcare workers. The study indicated a deficiency in employer-provided psychological support. Furthermore, it demonstrated that an increase in workload does not necessarily lead to an increase in employee expectations of recognition and appreciation. Overall, this study underscores the importance of comprehensive managerial strategies in maintaining organizational resilience and improving employee well-being during crises.

**Discussion:**

This study shows that resilience-based management—especially reliable PPE, testing, and clear internal communication—helps protect healthcare workers’ well-being during crises. Strengthening communication and psychological support before future emergencies remains essential. The findings echo existing research and lay groundwork for further work on healthcare resilience and staff well-being.

## 1 Introduction

Organizational resilience is a key element of a strategy for coping with adversity (1–10). Based on literature review conducted by Barasa et al. (9), organizational resilience is an organization’s capacity to keep achieving its objectives and to adapt positively under adversity, emerging from crises strengthened and enriched with new resources. Barasa et al. (9) specifies several factors that influence organizations’ resilience, which are material resources, preparedness and planning, information management, collateral pathways, governance processes, leadership practices, organizational culture, human capital, social networks and collaboration. In line with Duchek (8), organizational resilience is understood as an organization’s ability to anticipate potential threats, to cope effectively with adverse events, and to adapt to changing conditions. The 2025 scoping review, prepared by Ratliff et al. (11), synthesizes 97 papers (from period 1998–2023) and finds broad consensus on a core definition of organizational resilience in healthcare: the capacity of a facility or healthcare system to preserve its essential functions and achieve its goals when exposed to shocks such as pandemics, workforce shortages or financial stressors. Most studies treat resilience as a dynamic process comprising three dimensions: reactive (absorbing and rapidly adapting during a crisis), proactive (anticipating threats and planning in advance) and reflective (learning during and after a disruption to drive improvement). Some authors extend the concept further, adding a growth or transformational element that views crises as opportunities for innovation rather than a mere return to the status quo ante.

The report *Organizational and Employee Resilience* prepared by the Society for Human Resource Management (12) shows, organizational resilience is extremely important in the healthcare sector. The healthcare sector had difficulty coping with the challenges of the pandemic, second only to the government and education sectors. Of the 55 organizations surveyed, nearly half (49.1%) were less effective after the pandemic than before the health crisis. Organizations that maintained a similar level of effectiveness or recovered from a temporary decline accounted for 34.5% of the sample. On the other hand, 16.4% of the surveyed organizations emerged stronger from the pandemic. The report emphasizes that key factors influencing organizational resilience, such as crisis preparedness, response to the threat of a pandemic and actions taken during a pandemic, have a direct impact on employee well-being.

The literature has produced numerous concrete recommendations and tools intended to help healthcare leaders and teams strengthen organizational resilience. Drawing on a growing body of global empirical research, the WHO prepared a guidance *Building health system resilience to public health challenges* (13) translates organizational resilience theory into an actionable health-system agenda. It distills six attributes: awareness, mobilization, self-mitigation, integration, diversity and transformation, that collectively allow a health system to anticipate risks, absorb shocks, adapt operations and ultimately transform after crises. By mapping these attributes onto the WHO “building blocks” (governance, financing, workforce, information, medicines and service delivery), the guidance embeds resilience as a continuous improvement lens rather than a crisis-only concern.

Organizational and employee resilience are closely linked (14, 15). Employee resilience is defined as the ability to cope, adapt, and grow positively in response to dynamic and challenging environments, supported and facilitated by organizations (12). Organizational resilience enables employees to effectively overcome challenges, supporting adaptability, innovation and overall well-being, while increasing job satisfaction, productivity and retention rates, which translates into better performance of the organization itself (16, 17). The way individuals interpret and assign meaning to their work experiences greatly impacts their psychological well-being. The importance of well-being in the workplace has become more apparent, especially after the pandemic (18). Research on the relationship between organizational resilience and employee resilience is also emerging in healthcare. The results of Gröschke et al. (19) research are noteworthy in this area. Gröschke et al. found that resilience in German healthcare works on several linked levels. When individual employee resilience rises, so do the organizational resilience. In a crisis, the organizational resilience directly encourages adaptive behavior and partly explains how employee’s individual resilience turns into positive views of change. Ignatowicz et al. (20) compare three levels (micro-meso-macro) and emphasize that organizational resilience depends on the relationship with the individual level, i.e., the personal resilience of employees. Duncan (21) highlights: good communication, meaningful recognition, authentic leadership, collaboration, support for joint goals and vision, deployment of staff into crisis areas, supportive well-being strategies, developing strong social support. A study by Wrześniewska-Wal and Korporowicz-Żmichowska (22) shows that personnel policy reforms (e.g., expanding competencies) increase both the sense of agency among staff and the ability of institutions to adapt.

Implementing resilience-based interventions can be an effective strategy for maintaining a healthier and more resilient healthcare workforce (23) which, regardless of ownership, especially during a health crisis, is a unit that fulfills social objectives and not only pursues profit. If employees are unable to survive difficult periods healthcare providers will have difficulty providing services to patients.

The growing, yet still limited, body of research examining the relationship between organizational resilience and the well-being of healthcare employees (17–21), extant evidence reveals a consistent trend: organizations endowed with greater adaptive capacity report more favorable psychosocial outcomes among staff, including higher psychological safety and engagement and lower levels of burnout, anxiety, and depression (23–27). Despite a rapidly growing post-COVID-19 literature, there is still limited multi-level evidence on how organizational resilience translates into employee resilience and, ultimately, staff well-being, especially in Central and Eastern Europe. Additionally no empirical studies have been identified that examine how the organizational resilience factors highlighted by Barasa et al. affect the well-being of employees in healthcare organizations. There is a research gap that would show how individual interventions by organizations aimed at maintaining the functionality of healthcare providers during a health crisis have affected the well-being of healthcare staff. This study addresses this gap by examining the impact of a broad range of resilience-based interventions Resilience-based interventions, in this study, are defined as organizational actions that strengthen a healthcare institution’s capacity to cope with crises, such as ensuring adequate personal protective equipment and staff testing, clear risk communication, alternative care pathways (e.g., telemedicine) and psychosocial support.^1^ The study examines whether the selected factors were implemented effectively and efficiently enough to positively impact employee well-being. These factors fall into four categories, i.e., material resources, information management, collateral pathways, and human capital management [distinguished by Barasa et al. (9)].

The hypothesis tested was that resilience-based interventions positively influence employee well-being.

## 2 Materials and methods

### 2.1 Study design

Considering the whole cycle of the resilience process (8), this research focuses on the middle stage—coping—which is characterized by responding to current issues (concurrent action). The main concern of this stage is the development and implementation of quick and effective solutions. At this stage, social resources are considered a source of organizational resilience (8).

**Figure 1:**
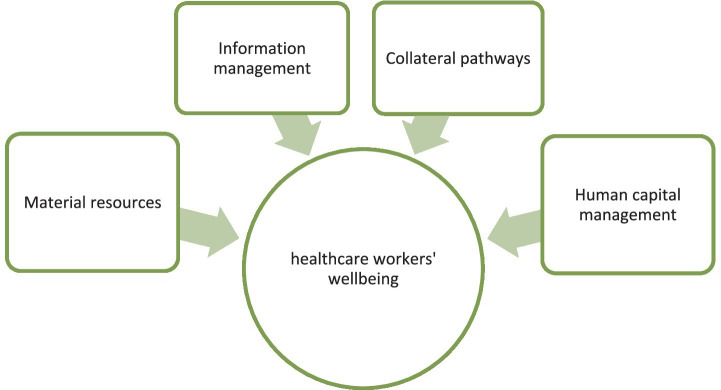
Conceptual research model—scope of the study. Source: Author’s own work.

This study follow Barasa et al. (9), who distinguished factors that influence organizational resilience. Additionally, we derive from Duncan (21), who indicated the factors that enhance the resilience in an healthcare organization. We combine their findings and propose a conceptual research model (Figure 1) that can help to examine how resilience-based management in healthcare organizations in Poland during the pandemic supported the well-being of their staff.

We assume that there are few management tools from factors of organizational resilience (9) different from human capital that significantly influence the workforce’s individual resilience and well-being. We define the inputs, i.e., the means that influence organizational resilience, and the outputs, which describe the healthcare workers well-being (Table 1).

**Table 1:** Setup of indicators and statistical methods used to verify the research issue.

Inputs (Organizational resilience factors)	Indicators considered in this study	Statistical methods
Material resources	Personnel Protective Equipment (PPE) availability	MMI test Chi-squared test of independence Proportional odds model for ordinal logistic regression
	Coronavirus test availability	
Information management	Action strategy communication	Proportional odds model for ordinal logistic regression Cochran Q test McNemar test
	Coronavirus knowledge communication	
Collateral pathways	Employees’ reallocation in the entity’s structure	MMI test Chi-squared test of independence
	New procedures that help to combat coronavirus	
	New procedures that help to maintain the accessibility to healthcare services	
Human capital management	Support from the employer	Cochran Q test McNemar test MMI test Chi-squared test of independence Logistic regression SPMI test
	Burden of duties	
	Employment contract type	
Output	Indicators considered in this study	
Well-being	Stress caused by the possibility of coronavirus infections	
	Sense of trust	
	Feeling undervalued	

The model proposed in the study analyzes the impact of four factors from a broader group identified by Barasa et al. (9) on healthcare workers’ wellbeing. The factors selected for analysis relate to activities within the organization and are: (1) Material resources, (2) Information management, (3) Collateral pathways and (4) Human capital management. They constitute inputs in the presented model. Each factor corresponds to several activities or phenomena that were examined in the healthcare workplace through our survey [following the Duncan (22) approach but as proposed by the authors of the study]. The activities quantified by the survey and the levels of the observed phenomena are indicators used in statistical analyses. We assigned two indicators from the survey to the Material resources factor: Personnel Protective Equipment (PPE) availability and Coronavirus test availability. We also assigned two indicators from the survey to the Information management factor: Action strategy communication and Coronavirus knowledge communication. The Collateral pathways factor was assigned the following indicators from the survey: Employees’ reallocation in the entity’s structure, New procedures that help to combat coronavirus, and New procedures that help to maintain the accessibility to healthcare services. The Human capital management factor was also assigned three indicators: Support from the employer, Burden of duties, and Employment contract type. The output of the presented model is Healthcare workers’ wellbeing, represented by the indicators Stress caused by the possibility of coronavirus infections, Sense of trust, and Feeling undervalued, as measured in the survey.

This study is a cross-sectional, online survey-based study addressed to healthcare professionals in Poland. The study focuses on healthcare workers as an essential element of organizational resilience. Healthcare personnel consist of all employed persons and volunteers who are potentially exposed to patients or their infectious materials.

A 39-item original questionnaire was developed and distributed using Microsoft (MS) Forms, 22 of which are used in this study. Additionally, the study also conducted in-depth interviews with the healthcare entities’ employees (1 doctor, 1 nurse, 2 diagnostic laboratory worker), which took place before preparing the questionnaire in order to indicate the most important issues which were then studied in the survey. The participants were recruited through websites and newsletters sent by doctors, nurses and midwives’ associations. The questionnaire study was conducted between August 21, 2020 and October 6, 2020. The survey was delivered in the Polish language. Participants were allowed to terminate the survey at any time they desired, and the survey was anonymous and confidential. It took 5–7 min to complete. An introductory paragraph outlining the study’s purpose was posted along with the survey. The survey’s purpose was to evaluate the participants’ reality and perceptions regarding the crisis management of personnel in a healthcare entity, as well as their subjective personal safety.

The questionnaire was divided into three sections, which were Section I (Metrics), Section II (Preparing a healthcare entity to functioning during a pandemic) and Section III (A sense of security). Section I consisted of three questions that collected information on the respondents’ employment, their position in the medical entity and its characteristics. Section II consisted of 13 items and was designed to evaluate access to Personnel Protective Equipment (PPE)^2^ and COVID-19 diagnostic tests, the appearance of new procedures and tasks, and the utilization of telemedicine. Section III comprised of six questions designed to evaluate the participants’ subjective perceptions about safety at work, confidence in management and sources of support, as well as a question about sources of information about COVID-19. The structure of the questionnaire was determined by the convenience of the participants, therefore the results are presented using a different division, corresponding to the aim of the study. An English translation of the questionnaire is provided in Appendix 1.

The research was conducted based on the answers to the questionnaire obtained from all types of treatment facilities: hospitals, outpatient clinics (including primary health cares, specialist outpatient clinics, dental offices, emergency services) and older adults home, which represented public, private and mixed ownership. The addresses of the questionnaire were medical professionals, administrative workers and diagnostic laboratory workers, with various forms of employment, in both managerial and non-managerial positions. The inclusion of all possible respondent groups was intended to counteract the selection bias in data collection. Their frequency measures are presented in Appendix 2. Furthermore, all 16 provinces of Poland differing in the COVID-19 infection rate were represented in the study. They can be combined into three regions, due to the number of new COVID-19 cases per million residents during the study period: (1) 694; (2) 1119; (3) 1965. Not all of the above-mentioned factors are investigated due to the focus of the study on internal factors of organizational resilience.

### 2.2 Statistical analysis

The general population of healthcare workers consisted of approximately 661.6 thousand subjects. A total of 488 respondents participated in the study, two of whom did not meet the inclusion criteria because they were not employees of healthcare entities during the pandemic. The final sample size included in the analysis was 486.

The factors of resilience setting in Table 1 determine using of 22 questions of the questionnaire corresponding to different types of variables: single response categorical variables (SRCV), multiple response categorical variables (MRCV) and ordinal variables. A variety of methods were used to analyze the obtained data. Descriptive statistics were applied to all variables to describe their basic characteristics. Additionally, depending on the variables’ types and purposes, different statistical tests and models were applied whose main goal was to determine relationships between factors of resilience and the resilience itself.

In order to examine the independence between two categorical variables when at least one of which can have multiple responses, the test for multiple marginal independence (MMI) and the test for simultaneous pairwise marginal independence (SPMI) were used. In the case of multiple responses traditional chi-square test for independence should not be used because the assumption that responses are made independently of each other is violated. The MMI test was used to examine the relationship between an SRCV and an MRCV and the SPMI test was conducted to analyze the association between two MRCVs. Both of them are extensions to the chi-square test for testing independence between single response categorical variables. For MMI and SPMI tests, the nonparametric bootstrap procedure with 100,000 resamples was used to estimate the sampling distribution of the test statistic. If the hypothesis of independence was rejected, the *post hoc* analysis was conducted using chi-square tests of independence. The individual tests were performed using the estimated sampling distribution of the individual statistics calculated in the bootstrap procedures. The *p*-values were adjusted using the Bonferroni method to account for the multiple comparisons issue.

A cumulative logit model with proportional odds was applied to model the relationship between an ordinal response variable with more then two categories and one explanatory variables. Regression models that consider the natural order of the dependent variable are simpler than multinomial models and also have greater power to detect existing relationships. Moreover, in each analyzed model, the proportional odds assumption was met. Furthermore, the logistic regression was used to model the binary response variable. For these models, the Wald test was used to check whether the effect of the explanatory variable is statistically significant.

In order to determine if the probabilities of success for three or more dependent binary variables are the same, Cochran’s asymptotic Q test was used. A binary response recorded for each category of the multiple response variable yields matched samples. Cochran’s Q test is an extension to the McNemar test for testing consistency of proportions for two related samples. In the case of rejection of the hypothesis of the equal probabilities by the Cochran test, the omnibus test was followed up by multiple pairwise comparisons. A *post hoc* analysis consisted of performing the McNemar exact tests with Benjamini-Hochberg correction for multiple testing.

Statistical tests were performed at the significance level of 0.05.

In each of the four categories of resilience, i.e., material resources, information management, collateral pathways and human capital management, selected statistical methods were used, depending on the problem being analyzed. The appropriate methods are listed in Table 1.

For all the above mentioned models and tests (except for the MMI and SPMI tests) assuming the significance level of 0.05, the test power of 0.8 and the effect size at medium level, the minimum sample sizes were determined. The maximum of the obtained sample sizes was 307, which is much smaller than the number of observations of the analyzed data set, which was 486. Since the sample size formulas are not provided for the MMI and SPMI tests, a bootstrap methods were chosen to perform these tests to ensure the power to detect alternative hypotheses (28, 29).

Less than two-thirds of the variables (13 out of 22) contained small missingness, ranging from 0.4 to 7% and one variable contained 26% missing values. The missing values were imputed by the random forest-based nonparametric imputation method. This method can handle any type of variables and does not require assumptions about the distribution of the data. Furthermore, it can cope with different types of variables simultaneously considering the arbitrary relationships between them.

**Figure 2:**
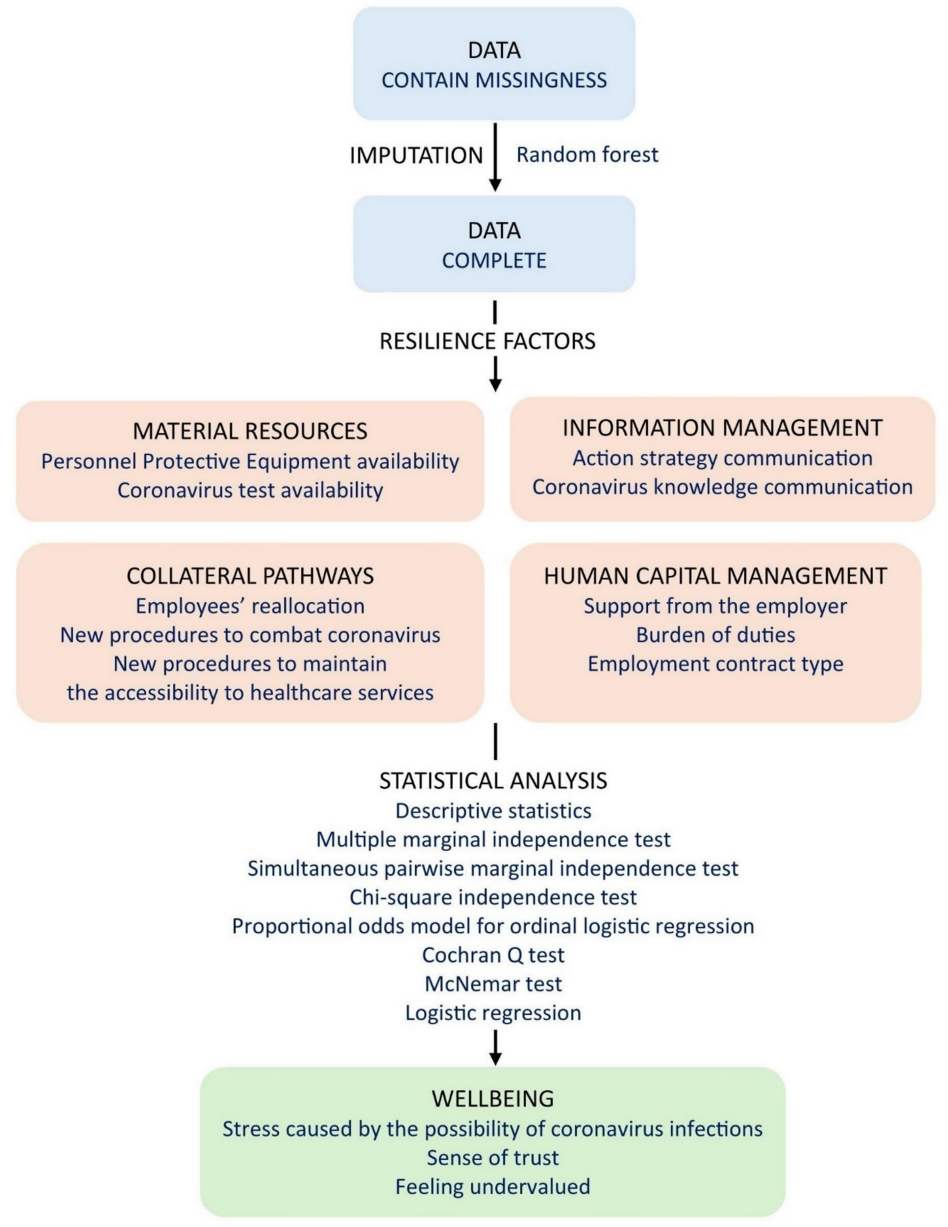
Data analysis process. Source: Author’s own work.

The data analysis process is presented in summary diagram (Figure 2). All analyses were conducted using R, version 4.0.3.

## 3 Results

Most of the respondents were females (79%), the participants were aged: 18–33 years (20%), 34–48 (36%) and over 49 (44%). These structures are consistent with the age and gender structure of the healthcare professionals population (30, 31).

An analysis of the variables and associations between them is consistent with the grouped factors of resilience setting in Table 1, i.e., material resources, information management, collateral pathways and human capital management. Data analysis included, among others, descriptive characteristics of all variables, number and percentage of respondents in each category. For some variables, these outcomes are presented in this section, while others are shown in the Appendix Table. The notation used in this section is: n, number of respondents; T, test statistic, p, *p*-value; p.adj, adjusted *p*-value; SE, standard error; OR, odds ratio; and CI, confidence interval.

### 3.1 Material resources

We analyzed the relationship between the availability of PPE and the level of anxiety related to the lack of appropriate PPE. Respondents were asked to tick what PPE they used in contact with suspected or confirmed COVID-19 cases and performing Aerosol Generating Procedures (AGPs). The results are presented separately for these groups. The MMI test indicates evident dependence for the group that had contact with COVID-19 cases and the *post-hoc* analysis shows a significant association between the coverall and anxiety. In the group that performed AGPs, the MMI test suggests independence of anxiety from the availability of PPE. In order to measure the different levels of protection offered by the PPE elements, values 1–3 were assigned to them: 1 for a fabric mask, gloves and visor, 2 for a standard surgical mask, gown or other equipment and 3 for eye protection, N95 or equivalent respirator mask and coveralls. The sum of the above values for the indicated items was used as an explanatory variable of the ordinal logistic model. The model allows us to confirm previous results in the group that had contact with COVID-19 cases. The availability of PPE had a significant but small effect. The odds of having a lower anxiety level increased by a factor of 1.05 with a one unit increase in the PPE score (OR = 1.05, 95% CI 1.01–1.09).

In this part of the study, the relationship between performing COVID-19 tests at work and anxiety over transmitting coronavirus to family members at home was also analyzed. For the variable “test,” three categories were considered: Yes, because I may have had contact with an infected person; Yes, they are performed as a screening test; No. The category “other” was removed due to responses having no clear relationship with the anxiety. The result of the cumulative logit model with proportional odds suggests a significant moderate association for screening tests. Being tested (except tests due to contact with COVID-19 cases) indicated that the odds of having the lower anxiety level was 1.55 times greater compared to not being tested (the reference category; OR = 1.55, 95% CI 1.08–2.23).

The detailed results of this part of the analysis can be found in Table 2.

**Table 2:** Results for the “material resources” factor.

PPE – Anxiety caused by its lack (contact with COVID-19 cases)				
MMI test	T = 150.25; *p* = 0.001***			
Post hoc to MMI test	*p*.adj			
The null hypothesis of the independence accepted (standard surgical mask, visor, gloves, other equipment, fabric mask, N95 or equivalent respirator mask, gown, eye protection)	from 0.121 to 1			
The null hypothesis of the independence rejected (coverall)	0.020**			
Proportional odds model for ordinal logistic regression	Estimate	SE	*p*	OR, 95% CI
PPE	0.048	0.021	0.025**	1.05, 95% CI 1.01–1.09
PPE – Anxiety caused by its lack (performing AGPs)				
MMI test	T = 85.99; *p* = 0.056*			
COVID-19 test at the workplace – Anxiety over transmitting coronavirus to family				
Proportional odds model for ordinal logistic regression	Estimate	SE	*p*	OR, 95% CI
Yes, because I may have had contact with an infected person	0.004	0.223	0.987	1.00, 95% CI 0.65–1.55
Yes, they are performed as screening tests	0.439	0.183	0.017**	1.55, 95% CI 1.08–2.23

*Statistically significant at the 0.10 level; ** at the 0.05 level; *** at the.01 level.

### 3.2 Information management

We analyzed the relationship between position and confidence in the management in terms of the procedures implemented to protect the staff against SARS-CoV-2 virus infection. Lead and non-lead positions were considered, which are significantly and strong related to confidence, according to the cumulative logit model with proportional odds. Respondents in a lead position are reported to have 3.33 times greater odds of having a higher confidence level than those in a non-lead position (the reference category; OR = 3.33, 95% CI 2.25–4.96).

We studied sources of information about the COVID-19 pandemic. Only 1% of respondents were not looking for information. Further analysis, including percentages in parentheses, concerns the participants who ticked any kind of information. The Cochran Q test results in strong evidence of significant differences in probabilities of sources selection. Multiple comparisons of 28 pairs using the McNemar test pointed out that, for 23 pairs, the probabilities were different. There are four groups of information sources about the pandemic, significantly differing in probabilities. In order of decreasing probabilities, they are (1) Authorities (59%), (2) Broadcast TV, radio (50%); the World Health Organization (WHO) and European Center for Disease Prevention and Control (ECDC) websites (46%), (3) Employer communications (35%); domestic medical websites (34%); social media (30%); self-government and associations (25%; this group is not homogeneous, which means that some of its items differ in probability), and (4) Other (3%).

The detailed results can be found in Table 3.

**Table 3:** Results for the “information management” factor.

Position – Confidence in the management				
Proportional odds model for ordinal logistic regression	Estimate	SE	*p*	OR, 95% CI
Lead position	1.204	0.197	0.000***	3.33, 95% CI 2.25–4.96
Sources of information about the COVID-19 pandemic				
Cochran Q test	T = 450.31; *p* = 0.000***			
McNemar test	p.adj			
The null hypothesis of the same probabilities accepted (5 pairs)	from 0.131 to 0.837			
The null hypothesis of the same probabilities rejected (23 pairs)	from 0.000*** to 0.013*			

*Statistically significant at the 0.10 level; ** at the 0.05 level; *** at the .01 level.

### 3.3 Collateral pathways

We examined information regarding the reallocation of employees within the entities’ structure. A small minority were redeployed to another area or specialty (18 out of 486), with 10 receiving training for their new roles. In this part of the analysis, we examined the relationship between new workplace COVID-19 prevention procedures and the anxiety of transmitting it to family members at home. Due to differences in procedures between the types of health entity, the results are presented for two groups, namely hospitals and other institutions (outpatient clinics, dental surgeries, emergency rooms and nursing homes). The MMI tests confirm the dependence for the hospital in contrast to the other entities, where this statement is not evident. However, the *post hoc* analyses for hospitals did not exhibit significant associations for any procedure. Thus, the overall association may be weak and should be interpreted with caution. In this part of the analysis, data on the isolation of healthcare workers as a way of reducing the transmission of the virus and on the procedures maintaining the accessibility to healthcare services were scrutinized. A minority of participants had to self-isolate due to having COVID-19 symptoms or contact with possibly infected individuals. New standards of diagnosing patients and telemedical solutions in outpatient work were introduced in most of the entities.

The detailed results of this part of the analysis can be found in Table 4.

**Table 4:** Results for the “collateral pathways” factor.

Employees’ reallocation within the healthcare entity’s structure		
Reallocation (*n* = 486)	Yes	18 (3.7%)
	No	468 (96.3%)
Area of reallocation (*n* = 18)	Intensive care unit/intensive medical care	1
	General medicine	3
	Department of Infectious Diseases	3
	Other	11
Satisfaction with the training in preparing for the new role (*n* = 10)	Not at all satisfied	2
	Partly satisfied	6
	Very satisfied	2
New procedures preventing the spread of the virus – Anxiety over transmitting the virus to the family		
Hospitals	MMI test	T = 132.25; *p* = 0.012**
	Post hoc to MMI test	p.adj
		from 0.215 to 1
Other entities	MMI test	T = 90.72; *p* = 0.064*
Isolation of healthcare workers and the procedures maintaining accessibility to healthcare services		
Isolation (*n* = 486)	Yes	59 (12.1%)
	No	427 (87.9%)
Change of standards of diagnosing patients (*n* = 486)	Definitely yes	136 (28.0%)
	Rather yes	136 (28.0%)
	Hard to say	82 (16.8%)
	Rather not	101 (20.8%)
	Definitely not	31 (6.4%)
Telemedicine as part of outpatient work (*n* = 486)	Yes	323 (66.5%)
	No	83 (17.1%)
	I do not know	80 (16.4%)

*Statistically significant at the 0.10 level; ** at the 0.05 level; *** at the .01 level.

### 3.4 Human capital management

Most of the respondents declared no or little psychological support from the employer. In the study, outcomes concerning support from various groups of people were also checked. Almost one in four respondents, namely 22%, did not experience any support. Further analysis, including percentages in parentheses, refers to the participants who received any support. The Cochran Q test states evident significant differences. Further multiple comparisons of 21 pairs using the McNemar test indicated that, for 20 pairs, the probabilities were different and there are six support groups differing in probabilities to a large extent. In order of decreasing probabilities, they are: (1) Relatives (76%), (2) Co-workers (59%), (3) Supervisors (39%), (4) Patients (23%) and Strangers (19%), (5) Patients’ families (11%) and (6) Other (3%).

Moreover, in this part of the analysis, we examined the relationship between the possible increase in the number of duties and the expectation to being appreciated. The majority of respondents (68%) reported more responsibilities, but the minority (26%) anticipated any form of recognition it. The MMI test suggests a weak dependence; however, the *post-hoc* analyses did not exhibit significant associations between the number of duties and any category of appreciation. In the case of treating “Appreciation” as a two-level response variable, with categories “Yes”–"No,” the logistic regression model results in significant and weak to moderate association with the number of duties. An increase in the number of duties by one level results in increased odds of appreciation by a factor of 1.49 (OR = 1.49, 95% CI 1.23–1.83).

88% of respondents pointed out the reasons behind the increase in the number of responsibilities and for them further data analysis is performed, including percentages in parentheses. The Cochran Q test indicated evident significant differences in percentages between the causes of the increased number of duties. Further multiple comparisons of the 28 pairs using the McNemar test indicated that, for 24 pairs, the percentages were different. There are four groups of reasons, which differ significantly in probabilities. In order of decreasing probabilities, they are (1) More procedures (77%), (2) Due to the fear of contagion, more time is taken up with standard activities (43%); Some staff do not come to work because they have young children (38%), (3) Some personnel do not show at work, because they are in a risk group (22%) and (4) Some staff do not come to work because they are ill (14%); Some staff do not come to work because they are extremely afraid of becoming infected (11%); More patients in advanced stages of illness (8%); Other (6%). The group is not homogeneous, which means that some of its items differ in probability.

Regarding the relationship between the type of employment and the expectation of the effort being appreciated due to the increased number of responsibilities, the SPMI test suggests independence (T = 47.73, *p* = 0.099*). When treating “Appreciation” as a two-level variable, with categories “Yes”–"No,” the MMI test also indicated no significant association between it and the employment type (T = 5.12, *p* = 0.381).

The detailed results of this part of the analysis can be found in Table 5.

**Table 5:** Results for the “human capital management” factor.

Level of psychological support from the employer (percentage in parentheses)											
Level	0	1	2	3	4	5	6	7	8	9	10
Number of respondents	160 (32.9)	59 (12.1)	53 (10.9)	37 (7.6)	21 (4.3)	72 (14.8)	12 (2.5)	18 (3.7)	15 (3.1)	8 (1.7)	31 (6.4)
Support from various groups of people											
Cochran Q test							T = 718.41; *p* = 0.000***				
McNemar test							p.adj				
The null hypothesis of the same probabilities accepted (1 pair)							0.145				
The null hypothesis of the same probabilities rejected (20 pairs)							from 0.0000*** to 0.0003***				
Amount of duties – Appreciation											
MMI test							T = 41.06; *p* = 0.028**				
Post hoc to MMI test							*p*.adj				
The null hypothesis of the independence accepted							from 0.117 to 0.926				
Logistic regression model		Estimate		SE			*p*		OR, 95% CI		
Amount of duties		0.402		0.101			0.000***		1.49, 95% CI 1.23–1.83		
Reasons for the increased number of duties											
Cochran Q test							T = 855.88; *p* = 0.000***				
McNemar test							*p*.adj				
The null hypothesis of the same probabilities accepted (4 pairs)							from 0.169 to 0.366				
The null hypothesis of the same probabilities rejected (24 pairs)							from 0.000*** to 0.020**				

*Statistically significant at the 0.10 level; ** at the 0.05 level; *** at the .01 level.

## 4 Discussion

In this study, we focused on internal managerial actions that ensure organizational resilience and examined their influence on various aspects of employee well-being. Practices such as ensuring access to personal protective equipment (PPE), offering routine screening tests, and providing clear internal communication emerged as particularly associated with lower anxiety levels among staff. Based on the theoretical assumptions of this study derived from the literature review, the key elements in maintaining organizational resilience are material resources, information management, collateral pathways, and human capital management as based on (9). These factors are key to assessing the resilience level of an organization during a crisis, as part of a broader crisis management framework—understood in line with Duchek (11) as encompassing the capacities of anticipation, coping, and adaptation. The ability to effectively transfer and implement resilience-enhancing solutions during a crisis serves as a foundation for building permanent, systemic governance structures.

The paradigm shift following the pandemic has been driven by increased awareness of the inevitability of future crises—not limited to, but including, climate change, antimicrobial resistance, and growing socioeconomic inequalities. This has led to a shift toward embedding these unpredictable challenges into formal organizational management systems. It requires expanding management control systems to enhance resilience-oriented adaptability, as emphasized by Weber et al. (9). Our findings about the resilience level of the organizations studied can be interpreted as the low-to-moderate maturity in resilience level, with the concentration on rather ad-hoc than institutionalized learning mechanisms. Analyzing the resilience maturity level of the healthcare institutions is the direction for future studies.

Resilience in healthcare is not solely an organizational or microeconomic issue—it is a systemic and societal one. The pandemic clearly demonstrated how insufficient resilience of healthcare institutions can negatively affect public health outcomes. One indicator of the Polish health system’s vulnerability was the drop in life expectancy in 2021, directly attributable to the pandemic. This challenge is exacerbated by worsening workforce shortages, reinforcing the need to develop structural solutions for enhancing resilience, which—as Hollaar et al. (17) argue—are integral to building healthy and sustainable workplaces.

As part of the analysis on the impact of material resources on organizational resilience, we found that PPE availability had a statistically significant, though modest, effect on reducing staff anxiety (OR = 1.05, 95% CI 1.01–1.09). More impactful was the provision of screening tests, which was associated with substantially lower anxiety about infecting household members (OR = 1.55, 95% CI 1.08–2.23). While this association suggests that screening may alleviate anxiety, it is also possible that staff who were less anxious to begin with were more likely to view management decisions positively or to report greater confidence in organizational measures. Therefore, the directionality of this relationship should be interpreted with caution, and future studies using are needed to clarify causal pathways. During the early pandemic, there was a global shortage of PPE (32, 33), including in Poland. We analyzed the concern about inadequate access to PPE among all healthcare workers and in a discrete group of workers who have direct contact with patient’s aerosols. The results obtained indicate that anxiety increases with less access to PPE among all employees of healthcare entities. However, the above relationship was not confirmed among medical professionals performing in direct contact with the patient’s aerosol, which might be explained by the fact that, in these situations, perceived anxiety is influenced not by the actual availability of protective measures but by the individual’s perception of the protection degree. Similar relationships were found for a group of nurses working during the SARS pandemic, where less anxiety was observed among personnel working directly with SARS patients, which could be explained, among other things, by a greater degree of competence of these personnel (34).

The feeling of fear among employees that they will infect their loved ones arises in pandemic situations (35). This study found that screening provision for staff by facility managers to determine whether they are infected with coronavirus reduced the staff’s fear (increase well-being) of bringing the virus home and infecting their loved ones. Experience from other countries confirms observations from Polish healthcare entities delivered by this study (36, 37). Yunias Setiawati et al. (38) additionally confirmed in their study that “a significant correlation was found between the level of (employee) resilience and anxiety experienced by healthcare workers during the COVID-19 pandemic. The lower the resilience, the higher the anxiety experienced.” Beyond individual-level impacts, the pandemic also triggered systemic responses in Poland aimed at enhancing organizational resilience through regulatory reform.

One of the direct consequences of the pandemic in Poland was a stronger emphasis on the quality of care processes and increased standardization. New regulations concerning quality management were developed, and the certification system for medical facilities was revised. All these actions aimed to compel healthcare facilities to implement integrated quality management systems and to introduce bottom-up, proactive process planning to ensure high-quality care, as well as improved monitoring of errors and deficiencies. At the central level, 60 quality indicators were introduced, categorized into clinical, patient-related, and managerial indicators.

While this regulatory shift can be positively assessed in terms of organizational resilience, it appears that the opportunity to simultaneously improve the working environment was not fully leveraged. This could have been achieved by introducing standardized safety indicators specifically for medical personnel. Such an approach would align with employees’ expressed views on the importance of safety measures in reducing workplace fear and anxiety. However, without institutional guarantees, even these expectations may be unmet in practice.

The lack of appropriate requirements regarding access to personal protective equipment may result in inadequate safety measures being maintained in individual healthcare facilities. The risk of such situations increases in the context of financial difficulties and the resulting temptation to cut costs. Many healthcare institutions are operating at a loss, have high levels of debt, and are facing liquidity problems. This highlights the need for institutional action to ensure an adequate level of material protection is maintained.

Interestingly, the association between PPE availability and anxiety was not confirmed among staff performing aerosol-generating procedures, suggesting that subjective perceptions of safety, professional competence, or exposure habituation may mediate this relationship.

In this study, we also examined the effectiveness of information management as a factor that builds organizational resilience. We assessed how the staff of Polish healthcare entities evaluated access to information and whether they understood the crisis management strategy implemented by the organization. Holding a managerial position was strongly associated with greater confidence in workplace safety procedures (OR = 3.33), highlighting a possible gap in effective downward communication within the organization. We examined the communication effectiveness of both the action strategy and the coronavirus knowledge. Existing literature supports the idea that effective internal communication during a crisis is vital for organizational resilience and well-being (39, 40).

We examined staff confidence in workplace safety measures set by management. The results revealed that managerial staff had more confidence than lower-level employees, suggesting potential internal communication issues. Inadequate internal communication heightens uncertainty and fear while diminishing organizational resilience. Duncan’s (21) research underscores the significance of internal communication in enhancing organizational resilience. During the SARS pandemic in Singapore, 93% of respondents considered workplace policies and protocols clear, emphasizing the benefits of effective communication (35). These findings indicate potential deficiencies in internal communication—particularly between management and front-line staff—which may contribute to uncertainty and reduced confidence in safety protocols. Given the limited use of employer-provided materials and the reliance on external sources, further in-depth research is warranted to assess and improve communication practices within Polish healthcare organizations.

Recent reviews confirm a growing body of research on healthcare communication, particularly in the context of patient-provider interactions and crisis response. However, the field remains fragmented, with notable gaps in empirical studies focused on internal institutional communication and staff well-being during public health emergencies (41–43).

For the comfort and safety of the work and the effectiveness of the therapeutic measures taken, continuous training of employees is also very important. Therefore, it seems reasonable that healthcare entities’ employees should have easy and quick access to the latest global information on pathogen control already in the workplace. The survey results showed that only 34.5% of respondents used materials prepared by their employer. Most of these individuals still used additional sources of information (159 of 166). The most frequently selected source of information was communications from central institutions such as the Chief Sanitary Inspectorate, Ministry of Health, National Health Fund. Secondarily, information was sought from WHO, ECDC, and media. The stream of information was, however, not managed by the employer. This finding coincides with broader efforts to improve institutional communication infrastructure.

Some actions were already initiated during the pandemic with the focus on developing healthcare information systems, particularly through digitalization aimed at improving data flow efficiency. These efforts have been reinforced at the European level by the European Health Data Space initiative, and at the national level by EU- and state-funded projects supporting clinical information system upgrades. As of mid-2024, all public healthcare facilities in Poland are required to implement internal quality and safety management systems, including mechanisms for disseminating safety standards and reporting adverse events.

In recent years, targeted interventions have been launched to strengthen leadership capacity in the sector. Their aim was both to enhance the operational efficiency of medical facilities and to shift organizational culture toward a more collective and inclusive approach involving medical staff. These efforts, carried out under the auspices of the Ministry of Health, included funding for MBA programs for management personnel and training programs—particularly in soft skills—for senior and middle-level managers, some of which were led by the authors of this study. There were also plans to introduce a requirement that hospital directors hold an MBA degree, although this regulation was ultimately not implemented. Currently, there is a lack of studies evaluating the outcomes of these efforts or their impact on improving communication in Polish healthcare entities.

The third aspect we examined concerning organizational resilience was collateral pathways. We investigated factors like employee reallocation, new coronavirus-related procedures, and procedures to ensure healthcare service accessibility.

During the first pandemic wave, there was a negligible number of employee reallocations in the entities’ structure (18 out of 486 respondents). Only eight of 10 the trained, reallocated individuals stated that they were satisfied with their preparation for their new roles (they attended training). The fact that another eight people were not trained is disturbing, although it is possible that these individuals already had the requisite competence. Nevertheless, it is important to note that training during a new pathogen threat is extremely important to healthcare personnel’s real and perceived safety (44).

Another indicator analyzed in the area of collateral pathways was the impact of the procedures implemented to protect against infection on the staff’s fear that they might infect their household members with the virus. The study found that the degree of staff concern depends on the procedures implemented. There is a need for more in-depth research to find what exactly procedures were crucial for well-being. One procedure was to isolate staff if there was suspicion of contact with an infected person but, during the first wave of the pandemic, only 12.1% of the respondents underwent isolation. It is important to note that respondents from entities with no screening tests pointed out in the additional submissions that what they missed most was employee screening.

While new procedures were implemented, in order to ensure the provision of medical services to non-COVID patients, standards of diagnosis and treatment were changed frequently according to 56% of respondents.

66.5% of the respondents reported that telemedicine solutions were implemented in their facilities. We expect that these alternative ways of providing medical services, apart from increasing organizational resilience, also improved the well-being of employees, because they reduced contact with patients and, consequently, the fear of infection. This assumption is further supported by studies among physicians, which emphasize both the perceived benefits and the organizational prerequisites for successful implementation. Studies conducted among physicians on the topic of remote care provision indicate a generally positive attitude (45). The benefits cited by medical personnel include easier data management, the ability to provide high-quality care to a broader group of patients, time savings for doctors, access to expert consultations, and opportunities for continuous learning. From the physicians’ perspective, the ease of conducting remote consultations is particularly important. Healthcare institutions should provide training for doctors in patient communication and the use of digital systems. This is crucial in the context of ongoing efforts to expand digital access to health data, as well as issues related to cybersecurity—which has emerged as a new threat affecting trust in digital health services.

Other of the managers’ actions included recommending that the staff reduce contact outside the workplace and locking or limiting the use of common spaces, such as social rooms to restrict the possibility of contact without the security of PPE. Similar actions were observed among healthcare managers during previous outbreaks (SARS) (34). Also noteworthy is the solution introduced in other industries, namely shift work of personnel where each shift consists of the exact same group of individuals (groups work alternately and do not meet). This approach prevents the uncontrolled spread of the virus, while safeguarding the continuation of services.

The last intra-organizational factor identified in the study that influences organizational resilience is human capital management. We examined the impact of various components, including employer support, burden of duties, and employment contract type.

For the first indicator analyzed, we asked respondents how they rated the psychological support from their employer and from whom they experienced any kind of support. Our findings suggest that psychological support from employers was perceived as insufficient by a significant proportion of healthcare workers. Specifically, 82.6% of respondents rated the support received at 5 or below on a 10-point scale, while 32.9% indicated having received no support at all. Given that ratings of 7 or above are typically interpreted in satisfaction research as indicators of a positive experience, scores at or below the midpoint may reflect unmet needs or dissatisfaction.

In the context of healthcare, where emotional and physical burdens were especially heightened during the pandemic, such ratings may signal deeper institutional challenges. These include strained communication with management, a perceived lack of recognition, or limited access to coping resources. While our study does not directly measure burnout, the widespread perception of inadequate support may represent a contributing factor—and highlights the need for targeted, systemic interventions to strengthen staff well-being.

Previous epidemics have demonstrated the long-lasting stress effects on medical personnel, similar to post-traumatic stress disorder (46–50). Therefore, it is crucial for medical facility managers to prioritize the physical and mental well-being of their staff during emergencies (46, 51, 52). Providing such support can bolster employee resilience, and involving psychiatric units in assisting both pandemic patients and staff is recommended (34).

Medical entities’ employees could count on various types of support from their loved ones (59.1% of respondents), co-workers (45.5%) and direct superiors (30.2%). Support from co-workers and direct supervisors can be associated with working well as a team. The positive influence of good team relations on organizational resilience is confirmed by the findings of Karlene Roberts’ research (53), which draws attention to mutual help, trust and openness among co-workers. It would be very interesting to continue researching what factors influence team building during a health crisis (39, 45, 54, 55).

The majority of the respondents (68%) reported an increased workload, mainly due to additional procedures (77%), lack of staff, either because they were in isolation or were left with young children or were in a high-risk group and were excused from coming to work, and increased time to perform standard procedures because of the increased level of care required in performing them (43%). Staff shortages due to quarantine were also felt during the SARS outbreak (34).

The discussed part of the study revealed additional threats to organizational resilience, such as employee absenteeism due to concerns about risky working conditions. Imai et al. (36) noted that approximately 20–30% of healthcare workers hesitated to work during future infection pandemics, regardless of their cultural background. This risk to organizational resilience was also observed in a US workforce study (56), which covered not only healthcare employees but also personnel from police departments, fire departments, public health departments, and correctional facilities. In our study, 53 respondents (11%) mentioned that some staff refused to come to work due to extreme contagion fears, leading to additional workload for others. Similar behaviors were observed in studies conducted in other countries: in Germany, during the H1N1 outbreak (57), in the US (58) or in Singapore (59). These international examples enrich our analysis. However, while international comparisons offer valuable insights into how healthcare systems responded to the pandemic, their interpretive value depends on contextual factors. Differences in pandemic timelines, institutional structures, and resource availability may have significantly influenced staff perceptions and organizational outcomes. For instance, Marmor et al. (60) noted that countries with centralized governance and early access to protective equipment likely fostered different levels of institutional trust than those with more fragmented systems or delayed interventions.

In this study, cross-national references were used to broaden the analytical lens; however, we acknowledge that system-level characteristics—such as the degree of decentralization, workforce capacity, and crisis communication protocols—critically shape organizational resilience. Future research should aim to integrate these contextual variables more systematically to enhance the understanding of which practices are transferable across health systems. Among those experiencing increased responsibilities, there is an expectation of reward for extra effort. However, it should be noted that some people are pessimistic about their extra effort being appreciated and this lack of expectation of reward is likely to result in frustration and, in the long run, professional burnout, which will translate into lower organizational resilience. Burnout syndrome is not just observed among healthcare workers in a pandemic and is associated with increasing workload, lack of control over the work environment and insufficient rewards (61–63). The survey results showed that the expectation of reward for extra effort is not dependent on the employment form (employment contract, contract, civil contract, internship). Furthermore, there is a risk that exhausted and frustrated employees will leave their jobs. Polish medical associations confirm these fears. Among reasons are: working conditions, opportunities for development, atmosphere and working hours, as well as salary level (64). The literature on the subject also points to such a threat (65). For next step of the research, it would be interesting to find out what are the most intensive motivators during the crisis for health workers, what are the factors preventing employees from burning out.

Among the resilience-enhancing practices studied, those with the clearest and most consistent associations with improved staff well-being were regular screening tests, high PPE availability (especially coveralls), and strong internal communication structures. By contrast, practices such as staff reallocation or isolation procedures showed weaker or inconsistent links to anxiety reduction.

## 5 Conclusion

The study identified links between the management of healthcare entities and the well-being of healthcare personnel, a key factor in organizational resilience. It also highlights the areas where resilience-based management in Polish healthcare institutions can be strengthened. It confirmed the predictions that the activities of healthcare providers in four categories, i.e., material resources, information management, side paths and human capital management, affect the well-being of healthcare professionals. The low ratings of employer communication and psychological support emphasize the need for more effective internal communication strategies and accessible mental health resources for staff, particularly during crisis situations. Our findings also indicate that staff anxiety depended not only on the presence of protective procedures, but also on how competently these measures were perceived to be implemented - pointing to the critical role of transparent communication, clearly defined responsibilities, and trust in leadership.

Based on these insights, we recommend improvements in three areas: (1) strengthening internal information flow to ensure alignment between management and staff, (2) embedding psychological support structures within crisis preparedness plans, and (3) evaluating the effectiveness of alternative service delivery methods such as telemedicine.

At the same time, the presented study is only a prelude to in-depth research on factors that seem to be crucial in every health crisis and can be prepared in the phase before the crisis occurs. Future research could be directed in assessing the resilience maturity using standardized models, or exploring the impact of digital health technologies on psychological safety and teamwork.

### 5.1 Study limitations

The present study is not immune to certain limitations. This study used an online questionnaire disseminated through mailing lists and professional platforms, which may have led to self-selection bias. Respondents who chose to participate could differ from the broader healthcare workforce in terms of digital literacy, engagement with pandemic-related discourse, or willingness to share views. As a result, the sample may overrepresent individuals with particularly strong opinions or experiences. Additionally, some categories of analyzed variables are of small size, e.g., employees’ reallocation within the healthcare entity’s structure, which limits generalizations. Some subtle experiences of healthcare workers (e.g., a sudden increase in the workload of laboratory diagnostics) may still require qualitative and quantitative analysis to fully understand the impact of organizational factors on the work of different professional groups.

Consequently, the findings cannot be generalized to the entire population of Polish healthcare workers, and conclusions should be interpreted with caution.

## Data Availability

The original contributions presented in the study are included in the article/Supplementary material, further inquiries can be directed to the corresponding author.

## Footnotes

^1^In the relevant literature (17), the term “resilience-based interventions” is often used for organized, deliberately designed programs whose primary aim is to enhance employees’ psychological resilience. In our study, we analyze various interventions across the domains of factors influencing organizational resilience identified by Barasa et al. (9).

^2^Personal Protective Equipment (PPE) refers to specialized clothing or equipment worn by individuals to protect themselves from exposure to hazardous substances or environments. It includes items such as gloves, masks, goggles, face shields, gowns, and protective footwear, and is commonly used in healthcare, industrial, and construction settings to ensure safety and reduce the risk of injury or infection.
