# Supplementary material for: Implementing resilience-based interventions for healthcare employee well-being: evidence from the pandemic crisis
Source: Front Public Health. 2025 Aug 25;13:1606595. doi: 10.3389/fpubh.2025.1606595 (PMC12414932; doi:10.3389/fpubh.2025.1606595)
Supplement: Supplementary file 1 [file Data_Sheet_1.docx]

Appendix 1

Managing the staff of healthcare providers

during the COVID-19 pandemic

Dear Readers,

we kindly request answers to questions regarding the conditions and comfort of the staff of medical entities during the COVID-19 pandemic. The survey is part of a

scientific study, the results of which will be published in a scientific journal.

We hope that the collected information will allow us to identify problems to be solved and good

practices that will contribute to your comfort level in the near future.

The survey was divided into four sections:

Part I: Metrics,

Part II: Preparing a healthcare entity to work during a pandemic, Part III: Sense of security

Part IV: Concluding Remarks.

The survey is fully anonymous. It is directed exclusively to employees of healthcare entities, including both medical staff and administration. Participation in the study is voluntary.

By participating in this study, you acknowledge that you are at least 18 years of age, work in a healthcare sector, and agree to participate in the study.

The survey is in the on line form, but a paper version is also available upon request.

The survey takes approximately 5-7 minutes to complete (number of questions: 30-38). You can stop filling out the survey at any time before clicking "submit" and exit the form without submitting a response. When you press "submit" the responses will automatically be saved to the database.

Thank you very much in advance for your time and sharing your experience!

If you have any questions, please contact us at:….

* Required

# Part II

Metrics

## Gender


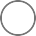
 Woman


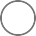
 Male


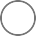
 I don't want to answer that question

## Age


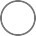
 18-25


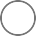
 26-33


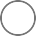
 34-40


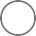
 41-48


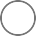
 49-56


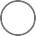
 above 56

## In which province do you work? *


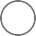
 DOLNOŚLĄSKIE


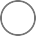
 KUJAWSKO-POMORSKIE


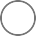
 LUBELSKIE


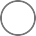
 LUBUSKIE


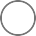
 ŁÓDZKIE


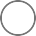
 MAŁOPOLSKIE


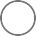
 MAZOWIECKIE


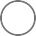
 OPOLSKIE


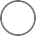
 PODKARPACKIE


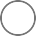
 PODLASKIE


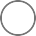
 POMORSKIE


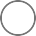
 SILESIAN


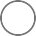
 ŚWIĘTOKRZYSKIE


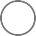
 WARMINSKO-MAZURSKIE


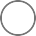
 WIELKOPOLSKIE


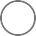
 ZACHODNIOPOMORSKIE


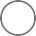


Other

## Type of treatment facility where you work *

*If you work in more than one entity, please select one and refer to the situation in that entity later in the survey.*


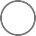
 Primary Health Care/family doctor


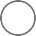
 Specialist outpatient clinic/clinic


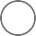
 Dental office


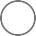
 Emergency service


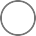
 Hospital


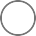
 Elderly home


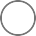
 Hospice


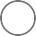


Other

## Hospital size


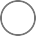
 up to 200 beds


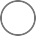
 201- 400 beds


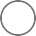
 over 400 beds

## The entity where I work is a facility:


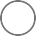
 Public


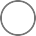
 Private


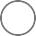
 Ownership is mixed


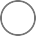


Other

## Position / role / distance from patient

*multiple choice question*


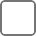
 managerial position


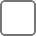
 medical professional (request to select in addition, below patient contact distance)


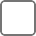
administrative worker (request to select in addition, below patient contact distance)


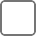
diagnostic laboratory worker (request to select in addition, below patient contact distance)


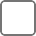
 an employee who has direct contact with the patient


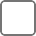
 an employee in contact with the patient at a distance of at least 1.5 m


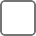
employee who has contact with "covid" patients (with suspected or confirmed viral infection)


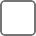
 an employee who is not in close contact with the patient


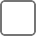
 I don't meet patients


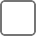


Other

## Form of employment


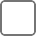
 Employment contract


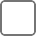
 Contract


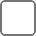
 Civil contract


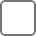
 Internship/practice


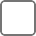


Other

## Have there been patients diagnosed with COVID-19 at your medical facility?


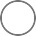
 YES


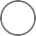
 NO


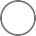
 I DON'T KNOW

## Did you participate in the care of a COVID-19 patient who died?


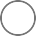
 YES


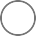
 NO

# Part II

Readiness of a healthcare provider to work during a pandemic

## What type of personal protective equipment did you have when you came in contact with a person infected or suspected of being infected with COVID-19?

*multiple choice question*


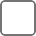
 fabric mask


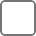
 standard surgical mask


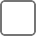
 gown


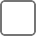
 eye protection


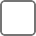
 N95 or similar mask


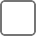
 protective suit


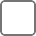
 gloves


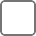
 visors


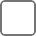


Other

## What type of personal protective equipment (PPE) did you wear while performing aerosol generating procedures?

*multiple choice question*


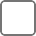
 I do not carry out the procedures associated with the generation of aerosol


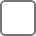
 standard surgical mask


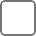
 gloves


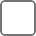
 gown


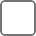
 eye protection


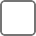
 N95 or similar mask


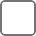
 protective suit


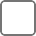


Other

## Have you been transferred to work in a different area than you normally work or in a different specialty than you have? *

*answer required*


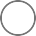
 Yes


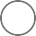
 No

## What area(s)/specialty(ies) have you been assigned to?

*multiple choice question*


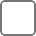
 Intensive care/intensive medical care unit


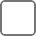
 Emergency medicine


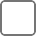
 General medicine


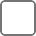
 Department of Infectious Diseases


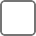
 I have not been transferred


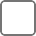


Other

## To what extent are you satisfied with the quality and quantity of additional training you received to adapt to your new role/position?


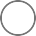
 No such training was organized


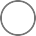
 I am not satisfied


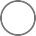
 I am partially satisfied


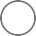
 I am very satisfied

## Have you been tested for COVID-19 in the workplace? *

*Answer required*


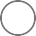
 Yes, because I may have had contact with an infected person


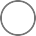
 Yes, they are performed as screening tests


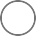
 No


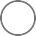


Other

## Did you exhibit symptoms typical of COVID-19 infection when you took the test?


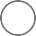
 Yes


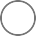
 No


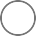


Other

## Have you been forced to isolate yourself because of exhibiting symptoms typical of COVID-19 infection or because of contact with potentially infected persons? *

*Answer required*


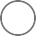
 Yes


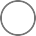
 No

## For how long (in days) were you forced to isolate yourself?


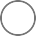
 1-3

4-6

7-9

10-12

12-14

over 14

Other

## Which of the following procedures have been implemented in your workplace to prevent the spread of the virus?

*Multiple choice answer*

All employees (including administration) must wear disposable masks

All employees (including administration) must wear disposable gowns

Only staff with patient contact wear disposable masks and gowns

Each patient and staff have a temperature measurement taken before entering the facility

Patients admitted to the inpatient unit have a coronavirus test

Personnel is screened and periodically tested for coronavirus

A ban on visits has been imposed

Restrictions were placed on movement between wards

The healthcare entity purchased and installed additional air and surface disinfection equipment in the premises of the healthcare facility

Each patient is initially interviewed for potential contact with COVID-19

Other

## To the existing procedures at my workplace, I would suggest adding:

1. Are you currently dealing with:

More patients

The same number of patients

Fewer patients

Hard to say

## Have the standards for diagnosing and treating patients at your facility changed in the era of the COVID-19 threat?

Definitely yes

Rather yes

Hard to say

Rather not

Definitely not

## During the COViD-19 pandemic, were telemedicine solutions introduced to work routinely with patients in the outpatient setting?

Yes

No

I don't know

## Do you have more responsibilities in the workplace during the pandemic period? *

*Answer required*

Definitely yes

Rather yes

Hard to say

Rather not

Definitely not

## Where do you see the reasons for your increased responsibilities:

*Multiple choice answer*

More procedures

The number of patients with advanced stage disease has increased

Some of the staff do not come to work because they are at risk and we had to take over their duties

Some of the staff do not come to work because they have small children and we had to take over their duties

Some of the staff do not come to work because they are extremely afraid of contagion and we had to take over their duties

Some of the staff do not come to work because they are ill and we had to take over their duties

For fear of contagion, I take more time with standard activities

Other

## Do you expect the extra effort to be recognized in the form of:

*Multiple choice answer*

Additional cash gratuity

Recognition/ praise

Chance of promotion

Will not be appreciated

Won't even be noticed

Other

## Do you observe an exodus of medical staff from your medical facility during a pandemic?

Yes

No

Hard to say

# Part III

Sense of security

## How often have you experienced anxiety due to lack of adequate personal protective equipment?

| 0 | 1 | 2 | 3 | 4 | 5 | 6 | 7 | 8 | 9 | 10 |
| --- | --- | --- | --- | --- | --- | --- | --- | --- | --- | --- |

never continuously

## Were you concerned that you might transmit the virus from your place of employment to your home and infect family members?

| 0 | 1 | 2 | 3 | 4 | 5 | 6 | 7 | 8 | 9 | 10 |
| --- | --- | --- | --- | --- | --- | --- | --- | --- | --- | --- |

definitely not definitely yes

## To what extent did you feel psychological support during the COVID-19 pandemic from your employer?

| 0 | 1 | 2 | 3 | 4 | 5 | 6 | 7 | 8 | 9 | 10 |
| --- | --- | --- | --- | --- | --- | --- | --- | --- | --- | --- |

Definitely no support Very strong support

## To what extent do you agree with the statement: "I have full confidence in the Management of the hospital/ambulatory in the procedures undertaken

to protect personnel from contracting the SARS- CoV-2 virus"?

| 0 | 1 | 2 | 3 | 4 | 5 | 6 | 7 | 8 | 9 | 10 |
| --- | --- | --- | --- | --- | --- | --- | --- | --- | --- | --- |

definitely not definitely yes

## To what extent do you agree with the statement: "I have confidence in my co-workers to follow established procedures to safeguard against contracting the SARS- CoV-2 virus"?

| 0 | 1 | 2 | 3 | 4 | 5 | 6 | 7 | 8 | 9 | 10 |
| --- | --- | --- | --- | --- | --- | --- | --- | --- | --- | --- |

Definitely not Definitely yes

## To what extent do you agree with the statement: "I have confidence in patients to follow procedures to protect themselves from infecting staff and other patients with the SARS- CoV-2 virus"?

| 0 | 1 | 2 | 3 | 4 | 5 | 6 | 7 | 8 | 9 | 10 |
| --- | --- | --- | --- | --- | --- | --- | --- | --- | --- | --- |

Definitely not Definitely yes

## To what extent do you agree with the statement: "My organization does not

has a coherent strategy for planning procedures of action in a crisis due to COVID-19 and each department takes care of itself"?

| 0 | 1 | 2 | 3 | 4 | 5 | 6 | 7 | 8 | 9 | 10 |
| --- | --- | --- | --- | --- | --- | --- | --- | --- | --- | --- |

Definitely not Definitely yes

## During the pandemic, you received support from:

*multiple choice answer*

Superiors

Colleagues

Patients

Patients' families

People close to you

Strangers

I have not experienced any support

Other

## During the pandemic, you experienced hostility/ostracism from:

*multiple choice answer*

Superiors

Colleagues

Patients

Patients' families

People close to you

Strangers

I have not experienced hostility/ostracism

Other

## What source of information do you most often use for the latest information regarding the COVID-19 pandemic?

*Multiple choice answer*

Internal communications prepared by my employer

Radio, television

Social media (Twitter, Facebook, LinkedIn)

Regulators (e.g., Chief Sanitary Inspectorate, Ministry of Health, National Health Fund, etc.)

Websites: World Health Organization (WHO) or European Centre for Disease Prevention and Control (ECDC)

National medical portals (e.g., Rynek Zdrowia, Termedia, [medonet.pl (http://medonet.pl)](http://medonet.pl/))

professional organizations, industry associations (e.g., Medical Chambers, Medical Associations)

Other

# Part IV

Concluding remarks

## Is there anything you would like to add that we left out of the survey?
