# Supplementary material for: Implementing resilience-based interventions for healthcare employee well-being: evidence from the pandemic crisis
Source: Front Public Health. 2025 Aug 25;13:1606595. doi: 10.3389/fpubh.2025.1606595 (PMC12414932; doi:10.3389/fpubh.2025.1606595)
Supplement: Supplementary file 2 [file Data_Sheet_2.docx]

| **Descriptive characteristics of the variables, number and percentage (in parentheses) of respondents in each category.** | | | | | | | | | | | |
| --- | --- | --- | --- | --- | --- | --- | --- | --- | --- | --- | --- |
|  | | | | | | | | | | | |
| **1. Type of treatment facility** | Hospital | | | | | | | | | 257 (52.9) | |
|  | Specialist outpatient clinic/clinic, dental office, emergency service | | | | | | | | | 174 (35.8) | |
|  | Elderly home, hospice | | | | | | | | | 55 (11.3) | |
| **2. Position** | Managerial position | | | | | | | | | 108 (22.2) | |
|  | Non managerial position | | | | | | | | | 378 (77.8) | |
| **3. Form of employment** | Employment contract | | | | | | | | | 361 (74.3) | |
|  | Contract | | | | | | | | | 73 (15.0) | |
|  | Civil contract | | | | | | | | | 25 (5.1) | |
|  | Intership/practice | | | | | | | | | 14 (2.9) | |
|  | Own business | | | | | | | | | 21 (4.3) | |
| **4. PPE in contact with suspected COVID-19 cases** | Fabric mask | | | | | | | | | 114 (24.5) | |
|  | Standard surgical mask | | | | | | | | | 305 (65.6) | |
|  | Gown | | | | | | | | | 255 (54.8) | |
|  | Eye protection | | | | | | | | | 148 (31.8) | |
|  | N95 or equivalent mask | | | | | | | | | 139 (29.9) | |
|  | Protective suit | | | | | | | | | 142 (30.5) | |
|  | Gloves | | | | | | | | | 408 (87.7) | |
|  | Visors | | | | | | | | | 307 (66.0) | |
|  | Other | | | | | | | | | 26 (5.6) | |
| **5. PPE during AGPs** | I do not carry out the procedures associated with the generation of aerosol | | | | | | | | | 339 (69.8) | |
|  | Standard surgical mask | | | | | | | | | 86 (58.5) | |
|  | Gloves | | | | | | | | | 132 (89.8) | |
|  | Gown | | | | | | | | | 87 (59.2) | |
|  | Eye protection | | | | | | | | | 81 (55.1) | |
|  | N95 or equivalent mask | | | | | | | | | 66 (44.9) | |
|  | Protective suit | | | | | | | | | 40 (27.2) | |
|  | Other | | | | | | | | | 22 (15.0) | |
| **9. COVID-19 test at workplace** | Yes, because I may have had contact with an infected person | | | | | | | | | 84 (17.3) | |
|  | Yes, they are performed as screening tests | | | | | | | | | 148 (30.5) | |
|  | No | | | | | | | | | 246 (50.6) | |
|  | Other | | | | | | | | | 8 (1.6) | |
| **11. New procedures preventing spreading of virus at workplace** | All employees (including administration) must wear disposable masks | | | | | | | | | 391 (80.5) | |
|  | All employees (including administration) must wear disposable gowns | | | | | | | | | 21 (4.3) | |
|  | Only staff with patient contact wear disposable masks and gowns | | | | | | | | | 124 (25.5) | |
|  | Each patient and staff have a temperature measurement taken before entering the facility | | | | | | | | | 363 (74.7) | |
|  | Patients admitted to the inpatient unit have a coronavirus test | | | | | | | | | 172 (35.4) | |
|  | Personnel is screened and periodically tested for coronavirus | | | | | | | | | 91 (18.7) | |
|  | A ban on visits has been imposed | | | | | | | | | 309 (63.6) | |
|  | Restrictions were placed on movement between wards | | | | | | | | | 214 (44.0) | |
|  | The healthcare entity purchased and installed additional air and surface disinfection equipment in the premises of the healthcare facility | | | | | | | | | 197 (40.5) | |
|  |  |  |  |  |  |  |  |  |  |  |  |
|  | Each patient is initially interviewed for potential contact with COVID-19 | | | | | | | | | 351 (72.2) | |
|  | Other | | | | | | | | | 21 (4.3) | |
| **14. More responsibilities in the workplace during the pandemic period** | Definitely yes | | | | | | | | | 186 (38.3) | |
|  | Rather yes | | | | | | | | | 147 (30.3) | |
|  | Hard to say | | | | | | | | | 60 (12.3) | |
|  | Rather not | | | | | | | | | 75 (15.4) | |
|  | Definitely not | | | | | | | | | 18 (3.7) | |
| **16. Expectations that the extra effort to be recognized in the form of** | Additional cash gratuity | | | | | | | | | 70 (14.4) | |
|  | Recognition/ praise | | | | | | | | | 51 (10.5) | |
|  | Chance of promotion | | | | | | | | | 2 (0.4) | |
|  | Will not be appreciated | | | | | | | | | 271 (55.8) | |
|  | Won't even be noticed | | | | | | | | | 186 (38.3) | |
|  | Other | | | | | | | | | 22 (4.5) | |
|  | | | | | | | | | | | |
|  | **0** | **1** | **2** | **3** | **4** | **5** | **6** | **7** | **8** | **9** | **10** |
|  | Definitely not | | |  |  |  |  |  | Definitely yes | | |
| **17. Anxiety due to lack of adequate personal protective equipment** | 69 (14.2) | 39 (8.0) | 59 (12.2) | 56 (11.5) | 31 (6.4) | 75 (15.4) | 34 (7.0) | 44 (9.1) | 40 (8.2) | 20 (4.1) | 19 (3.9) |
| **18. Anxiety over transmitting virus to family** | 27 (5.6) | 11 (2.3) | 28 (5.8) | 38 (7.8) | 19 (3.9) | 54 (11.1) | 30 (6.2) | 44 (9.0) | 59 (12.1) | 35 (7.2) | 141 (29.0) |
| **20. Confidence in the management in terms of procedures to protect staff against the virus** | 66 (13.6) | 35 (7.2) | 53 (10.9) | 54 (11.1) | 30 (6.2) | 92 (18.9) | 19 (3.9) | 28 (5.8) | 38 (7.8) | 22 (4.5) | 49 (10.1) |
